# Local experience of using traditional medicine in northern Rwanda: a qualitative study

**DOI:** 10.1186/s12906-021-03380-5

**Published:** 2021-08-13

**Authors:** Mengxin Tan, Yuko Otake, Teisi Tamming, Valerie Akuredusenge, Beatha Uwinama, Fabien Hagenimana

**Affiliations:** 1 London School of Hygiene and Tropical Medicine, London, UK; 2 University of Oxford, School of Anthropology and Museum Ethnography, Oxford, UK; 3 Conservation Heritage-Turambe, Ruhengeri, Rwanda; 4 Abavuzi Gakondo Rwanda Network - Rwandan Traditional Healers Association, Ruhengeri, Rwanda; 5 Institut d’Enseignement Superieur (INES-Ruhengeri), Ruhengeri, Rwanda

**Keywords:** Traditional medicine, Traditional healer, Community members, Illness

## Abstract

**Background:**

The popular use of traditional medicine in low-income settings has previously been attributed to poverty, lack of education, and insufficient accessibility to conventional health service. However, in many countries, including in Rwanda, the use of traditional medicine is still popular despite the good accessibility and availability of conventional health services. This study aims to explore why traditional medicine is popularly used in Rwanda where it has achieved universal health coverage.

**Methods:**

The qualitative study, which included in-depth interviews and participant observations, investigated the experience of using traditional medicine as well as the perceived needs and reasons for its use in the Musanze district of northern Rwanda. We recruited 21 participants (15 community members and 6 traditional healers) for in-depth interviews. Thematic analysis was conducted to generate common themes and coding schemes.

**Results:**

Our findings suggest that the characteristics of traditional medicine are responding to community members’ health, social and financial needs which are insufficiently met by the current conventional health services. Participants used traditional medicine particularly to deal with culture-specific illness – *uburozi*. To treat *uburozi* appropriately, referrals from hospitals to traditional healers took place spontaneously.

**Conclusions:**

In Rwanda, conventional health services universally cover diseases that are diagnosed by the standard of conventional medicine. However, this universal health coverage may not sufficiently respond patients’ social and financial needs arising from the health needs. Given this, integrating traditional medicine into national health systems, with adequate regulatory framework for quality control, would be beneficial to meet patients’ needs.

## Background

The World Health Organisation (WHO) defines traditional medicine as “the sum total of the knowledge, skill, and practices based on the theories, beliefs, and experiences indigenous to different cultures, whether explicable or not, used in the maintenance of health as well as in the prevention, diagnosis, improvement or treatment of physical and mental illness” [1]. In African settings, this includes, but is not limited to, local herb medicines and products or indigenous healing practices [2]. The application of traditional medicine remains popular in sub-Saharan Africa, with use among the general population being reported with prevalence rates from 4.6 to 94%, dependent on the setting [3]. In 2004, the WHO African Regional Office has developed tools and guidelines for improving and standardising the practices [4].

A systematic review on why people use traditional medicine in Sub-Saharan Africa demonstrates several important reasons for its continued popularity [3]. One common reason is the alignment of traditional medicine with sociocultural, religious and spiritual values. African traditional medicine has been considered as providing meaning for illness and suffering [5]. Secondly, people have trust and confidence with traditional healers to share their personal secrets. Trust with the traditional healers is often achieved through recommendation by trusted elders, friends and family. Additionally, literature has found that low cost, flexibility of payment, as well as the accessibility drives the use of traditional medicine [3].

Recent studies have discussed the role of traditional medicine in achieving universal health coverage (UHC) [6, 7]. A critical component of UHC is improving the access to health coverage. Generally, traditional medicine is regarded as more accessible, affordable and acceptable to the local community members. It could therefore contribute to the attainment of UHC [6].

### Traditional medicine and universal health coverage in Rwanda

Traditional medicine in Rwanda and neighbouring countries in the African Great Lakes Region includes herbal prescriptions, animal parts, minerals and spiritual narratives to treat illnesses [2, 8–10]. Among various health conditions that are treated by traditional medicine in the Great Lakes Region, ‘poisoning’ (*uburozi* in Kinyarwanda) is the most common illness for which local people seek a traditional medicine treatment [11]. This is a culture-specific illness, which refers to the local variants of psychiatric and somatic symptoms that are considered to be recognizable illness only within a specific society or culture [12, 13]. “Poisoning”, also interpreted as “sorcery” or “witchcraft”, is not always a literal poisoning of eating or drinking a substance which contains a poison. Instead, in many societies in the African Great Lakes Region, “poisoning” is understood to be enacted by distantly sending magical substances through air, placing them on the path of a person, or within close proximity through shaking hands among others [9]. Underlying the local understanding of health and illness in the Great Lakes Region, Taylor [8] documented the concept of flow and blockage observed in Rwanda. Images of blocked or excessive flow of bodily fluids permeate symbolic constructs related to health, notions of the self, and ideas about the human body. Schierenbeck and other colleagues [14] additionally suggest that traditional perceptions of mental illness are combined with Christian conceptions of demonic possession and witchcraft in Rwanda. Local people believe for example that mental illness is the result of a devil or spirit possession, so they turn to traditional healers.

Rwanda is well-known as a country that has achieved UHC through the wide implementation of the community-based health insurance (CBHI) [15]. The CBHI covers more than 90% of the population [16]. Despite the fact that Rwanda has universal coverage of conventional health services with over 90% of population covered, traditional medicine remains popular. Previous research has described the use of traditional medicine for treating different diseases, including skin diseases, pregnancy, and liver problems in Rwanda [17–19]. However, the reasons for which people use traditional medicine, even when conventional health services are accessible, available and affordable under UHC, has not been studied. As traditional medicine is perceived as an important community-based healthcare resource [20], it remains pivotal to understand the experience of using traditional medicine from the perspective of local communities that rely on this resource. Thus, the aim of this study is to understand the local experience of using traditional medicine in Rwanda – a Sub-Saharan African country which has achieved universal health coverage,and explore why traditional medicine continues to be needed and utilised by the local community. By exploring this, the study will contribute to the development of local and global policies of integrating traditional medicines into the national health systems.

## Methods

This qualitative research was informed by a rapid ethnographic study design using in-depth interviews and participant observations to respond to the study aim.

### Research site

The research took place in the Musanze district in the Northern Province of Rwanda. Musanze district was selected as the research field due to the popular use of traditional medicine among the local people. In particular, the area of Kinigi in Musanze is well-known as the core location for traditional healers in the country [21], which represents traditional medicine application in northern Rwanda.

### Research participants and sampling strategy

A total of 21 participants (age range: 27–84 years) took part in the in-depth interviews. The sample consisted of 6 traditional healers (who are acknowledged by the community members and well-known for traditional medicine practices in the community) and 15 community members who live in Burera, Bushozi, Musenyi and Rugali villages (Table 1). They were approached through the fourth author’s networks in different villages through word-of-mouth. The first author and the fourth author sought the approval from village leaders from each village for this study. Initial sampling was conducted to maximise a variety of characteristics representing the research population [22]. It gradually involved neighbouring areas to include a maximum variation of participants’ characteristics, including age, gender, occupation, socio-economic status, and home village, in order to facilitate analysis of experience of using and providing traditional medicine. Secondly, in order to do theoretical sampling, the first author concurrently analysed the initial data during the participant recruitment phase, which determined future participant recruitment. After that the sampling shifted to theoretical sampling, through which relevant data was sought in response to analytical questions and preliminary hypotheses arising from the initial data analysis. The theoretical sampling terminated when the data fully tested emerging hypotheses, called reaching the “theoretical saturation” [23].

**Table 1:** Characteristics of research participants

	Number (Total 21)
**Gender**	
Female	13
Male	8
**Age**	
21–40	5
40–60	7
Over 60	9
**Occupation**	
Farmer*	12
Traditional Healers*	6
Small business owners	2
Non-governmental organisation officers	1
Others (cleaner, teacher, government officer)	3
**Education level**	
Bachelor degree	4
Secondary School	7
Primary School	5
Never attend school	5
Socio-economic status**	
Middle income	14
Low income	7

*There is an overlap of occupations with farmer and traditional healers. Three farmers expressed they practiced traditional medicine practice, but their main income is still from their farming work

** Socio-economic status is self-reported by the participants based on the governmental standards

### Data generation and analysis

Data were generated through in-depth interviews and participant observations after obtaining informed consent from village leaders and research participants themselves. Participant observations focused on the health-seeking behaviours for traditional medicine and the treatment processes by which community members apply traditional medicine. The first author wrote field notes based on her living experience in the villages, community observations and daily conversations with community members to enrich her understanding of the local context. The first author conducted in-depth interviews with the interpretation and contextual understanding support from the fourth author who is from the local community of Musanze district between June 2019 and July 2019 over a period of four weeks. Member checking was done with the fourth author (local research assistant) continuously and fifth author (a traditional healer) to verify interpretation and themes from the data analysis, in order to ensure the trustworthiness of the result interpretation.

The first author initially developed interview topic guides with the second author to explore the research aim. After piloting the initial topic guides with two participants, the first author revised the topics guides through close discussions with the fourth author who is from the local community, in order to ensure that the questions were contextually appropriate and easily understandable for the participants. Two versions of the topic guides were developed. One was used for community members to explore their personal experience of seeking and using traditional medicine (Table 2). The other version was used for traditional healers to understand their traditional medicine practices (Table 3). The questions were loosely structured and conversational to allow flexibility of discussion and a variety of perspectives to emerge. All interviews took place in the participant’s home or a location where the participant felt comfortable by their own choice. Each interview lasted on average 30–40 min. All the interviews were audio-recorded and were transcribed verbatim. Two professional Kinyarwanda-English translators translated the interview transcripts into English with double-checking the translations.

**Table 2:** Interview Topic Guide for Community Members

**Main questions for community members**	
Q1: Please tell me in detail about one of your visits to a traditional healer.	
- How did the traditional healer diagnose your problem?	
- What treatment you were given?	
- What happened?	
- What was the health outcome? / How long did it take to recover?	
- Have you visited a hospital for the same problem?	
Q2: For what health problems will you look for traditional healers?	
Q3: How did you find traditional healers?	
Q4: How did the traditional healer charge you?	
Q5: Have you experienced any side effects of using traditional medicine?

**Table 3:** Interview Topic Guide for Traditional Healers

**Main questions for traditional healers**	
Q1: Please tell me how do you become a traditional healer?	
Q2: What health problems can you treat with the use of traditional medicine?	
Q3: Can you give a recent example of how you treat a patient in detail?	
- How did you diagnose the problem?	
- What treatment did you provide to patients?	
Q5: How do people find you?	
Q6: How do you charge your patients?	

Thematic analysis was conducted to generate coding schemes and themes. Initially, the open coding summarised segments of text line by line. Then, axial coding grouped the fractured codes to develop themes. QSR Nvivo 12.5.0 was used to assist the whole analysis and coding process.

## Results

Three main themes were identified through thematic analysis to answer the research question that why community members use traditional medicine despite Rwanda’s achievement of UHC. All main themes and sub-themes described the ways in which traditional medicine meets community’s specific needs which were insufficiently addressed by the conventional health services. Theme 1 explains the diagnoses, treatment and relationship characteristics of traditional medicine that respond to patients’ health, social and financial needs. Theme 2 illustrates the specific illnesses that traditional medicine is perceived to treat better than conventional health services from patients’ point of view. Theme 3 shows the ways in which traditional medicine responds to patients needs that were not sufficiently met by the conventional health services.

### Theme 1: Traditional medicine meeting health, social and financial needs

#### Person-centred diagnosis & treatment understanding and engagement

Participants reported that traditional medicine responds to their health and social needs through its diagnostic and treatment characteristics including person-centred and patient engagement approaches. Participants explained that the traditional healers diagnose their health problems by observing, touching and listening. Specifically, traditional healers diagnose the health problems with visible external symptoms, such as skin problems and eye problems, by looking and touching the body parts where the illness symptoms are present. For health problems without distinct external symptoms such as stomach ache and headache, traditional healers’ diagnosis is based on the patient’s description. In this case, the patient’s description is vital for diagnosis. Thus, traditional healers spend time carefully listening to patient’s description of the illness symptoms and health complaints to make diagnosis.

“So I went, all the traditional healer did, was just taps to my baby’s body. The whole body, you know, with hands.” (P-02, community member )

“I have to consult my patients. For visible illnesses, I can diagnose symptoms but for invisible diseases, the patients tell me how they are suffering. When I find out that I can treat it I openly say it and give them medicine depending on what they have told me.” (P-09, traditional healer)

The components of traditional medicine depend on the illness and patient’s condition.

Most commonly, traditional medicines are made of natural products, such as herbs, minerals (e.g. soil, clay, rock), animal parts (e.g. cow ghee, oil), foods (e.g. garlic, onion, salt). The most popular forms of traditional medicine are those based on herbs. Traditional healers prepare the treatment materials by mixing several ingredients tailored to each patient’s illness condition.

*“I was feeling the pain in the legs and feet. I was scratching them because they were very painful … . They (traditional healer) gave me some medicines to boil in water and mix it with rosemary and I drank that liquid three times a day for fifteen days. Also, I was eating onions and carrots mixed with lemon which were taken with food three times every day for a period of fifteen days “ (P-12 , community member)*

The traditional healers sometimes engaged the patient in the treatment preparation process. For example, in one case, after listening to the patient’s illness history of asthma, the traditional healer prepared medicine by squeezing different herbs together and boiling the herb with water, and explained to the patient that s/he used a type of herbal plant which can help the patient breathe smoothly after drinking it. The patient witnessed the process of the medicine preparation and understood the ingredients of traditional medicine through the conversation with the traditional healer. Moreover, some community members showed good understanding and knowledge of traditional medicine materials they were given. They described specifically the name of herbs and other materials that used for treating their illness.

*“I suffered from coughing and I was given the medicine called Rumex bequaertii (nyiramuko) which was to be chewed... Also, when you are suffering from stomach-ache, there is a medicine called “gitinywa” used to treat intestinal worms. Coughing is also treated using small papaws which are not ripe yet. They have to be chewed or baked in fire and squeezed for extracting some water.”(P-07, community member).*

#### Closeness and convenience within social networks

Traditional healers are easily accessible through the local networks by word of mouth. This also contributed to meet the patients’ social and financial needs. Community members share the information of where the traditional healers live, which traditional medicine practices they do and what illnesses they are able to treat. Patients with a health problem can be guided to a traditional healer by someone who has had the same problem and who has previously been treated by the traditional healer.

*“Actually they (traditional healers) are known. They move from place to place, and there are those who stay in their homes. We actually know where they live, and they are known for practicing traditional medicine. You meet them there and tell them about your illness. “(P-07, community member).*

Traditional healers are living in the shared communities with patients and aim to maintain good relationships with them. Therefore, traditional healers charge the community members flexibly by considering various factors, including the type of illness, the health outcomes after receiving treatment, and the financial situation of the patient. Some community members reported that the traditional healers had charged them an initial fixed amount of money after consultation. The first consultation fee was reported to range from 2000 FRW to 5000 FRW (equal to $2.02 to $5.05 on 20th December 2020). Subsequently, when patients get better, they pay an additional fee or bring a gift to show their appreciation. Although most traditional healers did not require patients to pay immediately after their recovery, patients were aware to show their appreciation to traditional healers based on their financial capacity, following the social norms of reciprocity. Both community members and traditional healers reported that traditional medicine can be free of charge. The free-of-charge treatment occurs when the patient is financially vulnerable or has a close relationship with the traditional healer.

*“It is like going to borrow a bull and they don’t charge you immediately, but when your cow calves, you will give the milk to him/her.” (P-08, community member)*

*“I often do it in order to simply help people. For example, there are people I do give traditional medicines for free of charge, and as today I have got a cow through ‘Gira inka program‘ (One cow per poor family program), these other people give me the grass for it as a sign of recognition of what I have done for them.” (P-09, traditional healer)*

However, the treatment of traditional medicine can be expensive, as an example of the extreme case a participant shared about a past experience of selling a land in 2003 in order to obtain a traditional spiritual healing treatment.

*“then he (traditional healer) told us how he will charge us. And it was so expensive that time for us, because we were unable to pay the money. We sold the land for 80,000 FRW (equal to $81.81 on 20th December 2020). See in 2003, that was a lot money.” (P-02, community member)*

### Theme 2. Traditional medicine meeting needs for treating specific illnesses

#### Treating minor illness, somatic pains, skin problems and hepatitis

Participants reported that they used traditional medicine to treat minor illness, including coughing, sneezing and minor injuries (e.g. wounds). Traditional medicine was handy in community members’ own gardens and traditional healers were living in close proximity within the community. Therefore, traditional medicine and traditional healers were perceived to be a convenient health resource in the community for dealing with minor illnesses.

*“I have been busy working for myself. When I felt sick of coughing, flu, I used Tetradenia riparia (umuravumba) instead of going to health centre/hospital.” (P-03, community member)*

Furthermore, community members seek traditional medicine for treating other specific illnesses including somatic pains, stomach problems, skin problems and hepatitis. These illnesses are perceived to be treated or relieved by the use of traditional medicine.

*“I felt much pain at the level of arms and went to traditional healers for treatment. I still have some of their medicines. I rubbed the medicine on my arms and felt relieved” (P-21, community member)*

*“I, too, suffered from a skin disease but now I feel better. I can even say that I have completely recovered. So, traditional healers are really effective.” (P-11, community member)*

#### Treating culture-specific illness: Uburozi (poisoning)

More importantly, participants commonly reported that there is a culture-specific illness which only traditional healers are able to treat, namely, ‘*uburozi* (poisoning)’. *Uburozi* (poisoning) is believed that can be caused by harmful substances or bad spirits that transmitted by an unknown/ suspected person or ancestors to make the target person sick directly. Poisoning is believed to be transmitted through different means, with or without direct contact; such as within close proximity by shaking hands, put in the food or drink, or put on the road where the targeted person will pass by.

*“Poison, apart from the ancient times, there are people who still have it but I can’t tell you where people get it from … . For example, there used to be men who practiced witchcraft in the night, who spent their night moving from place to place, but there also used to be women who poisoned kids or other women through food.” (P-12, community member)*

*“You sometimes hear people saying that they don’t know where people get it from. It is also said that people go outside the country to get and sell it. It is therefore served with food or drinks without being aware of it. People realized that there was poison when they had already eaten it. Also, poison can be deposited on the way, for example, or at the door or thrown inside the house. When you pass over it, it makes you. … ..there was poison that was thrown here in the house and in the corridor. We had been passing over it when going to bed.” (P-13, community member)*

Mental illnesses, somatization and fever are perceived as symptoms of ‘*Uburozi* (poisoning)’. The symptoms of mental illness included abnormal behaviours and emotions. When community members attributed the symptoms of mental illness to poisoning, they sought traditional healers for providing treatment to alleviate the symptoms.

*“ The way it started, she became quiet. We couldn’t tell what was wrong. And finally she end with by shouting, screaming, telling the stories with non-sense. One neighbour told that she was just having mental troubles and she was mentally troubled … . finally my aunt decided to go see a traditional healer who could help with the situation … the traditional healer said she was poisoned.” (P-02, community member)*

The other commonly reported symptoms of *uburozi* were somatization (headache, leg pain, stomach-ache) and fever. For example, community members reported that they experienced jealousy-caused “poisoning” which brought unusual pain on their feet and legs, shoulders, stomach, head, and spread further in the whole body. Participants also reported that poisoning can make people feverish, weak and unable to stand or walk. By any means, it is believed that jealousy-caused poisoning makes the targeted person unable to work and achieve anything due to the severe body pain, fever and other illness symptoms.

*“I was feeling pain here in the heart, in the stomach, at the hip joint, in the back, in the face, and I felt as if my brain was removed. That is how you feel pain when you are suffering from poisoning.” (P-20, community member)*

*“The symptoms I was displaying included stomach ache, and I could not walk because I could feel sharp piercing things in my legs and many endless piercing sore. I couldn’t even hold anything on my hands because they were burning.” (P-11, community member)*

Community members hold different beliefs of who used the poisoning on them or their relatives. The suspected “poisoner” can be someone close to the victim (e.g. working colleagues, neighbours), or it can be an unknown person who is believed to have administered the harmful substance. Even though the victims of poison do not know who exactly poisoned them, their neighbours, friends, traditional healers and themselves believe that someone poisoned them to threaten their life due to jealousy of their harvest, income, or high achievements.

*“I know someone who poisoned me … … When people saw that I had cows and I was starting to build my beautiful house, supplied with water and electricity, while still growing cows, they felt jealous of me. They are still in pursuit of my wife threatening to kill her because they finally want to make me a widower.” (P-21, community member)*

Traditional healers diagnose and treat poisoning through attentive listening and treat it by prescribing herbs, oil and mineral powders for aromatic use, massage, purification and protection rituals (e.g. cleaning the body with herbs for purification, rubbing the head and body with herbs for protection) and provide culture-specific explanations of the aetiology of illness symptoms (i.e., poisoning). It is widely believed that poisoning (*uburozi*) can only be treated by traditional healers instead of medical doctors.

*“The reason why we can confirm that traditional medicine exists and is helpful is that when someone gets poisoned or bewitched, they can’t go for modern medicine first. Instead, they go to the traditional healers who will give them some medicines that will help them spew the poison.” (P-04, community member)*

Within these two sub-themes, importantly, a few safety issues of taking traditional medicine were reported. For example, a participant narrated about their neighbour who was given traditional medicine without precise measurement instruction from a traditional healer, which therefore resulted in overdosing. Also, one participant reported that a lack of standard expiry date on traditional medicine provided by the traditional healer can have a negative effect on the use of herbs, because there is no refrigerator to store the herbs in their homes.

### Theme 3: Traditional medicine meeting needs which are not sufficiently met by the conventional health services

#### Used as a first-aid treatment when conventional health service is not available

Based on the reported experience, traditional medicine is particularly useful when the patient is far from the hospital but still needs first-aid. One participant gave the example of his brother who had been bitten by a snake in a forest which was a long way from the hospital. They quickly visited a local traditional healer who removed the venom by lighting a match on the place where the snake had bitten him. Another participant reported his experience of getting wounded in the forest. He subsequently collected herbs in the forest to cure the wound instead of going to the faraway hospital.

*“For example, if you suffer an injury in the forest and you realize that the medical help will be far, you can put on it Bidens pilosa (inyabarasanya) before you can go to the hospital.” (P-05, community member)*

#### Used as an alternative and/or complementary treatment to conventional health services

Community members visited traditional healers because the treatment in hospitals failed to cure their illness. When participants found that their symptoms did not get better, or even worsened in hospital, they turned to traditional healers.

*“Every time the child fell sick, I was told to take her/him to hospital. I found out that the situation was deteriorating to an extent that s/he was getting out of control. Yes, I took the child to the traditional healers, s/he was under treatment for a period of three months before s/he got healed” (P-11, community member)*

In some cases, community members visited traditional healers because medical doctors referred their patients to traditional healers for the culture-specific illness -“poisoning”. This occurs when the doctors themselves recognise that they are unable to treat the illness symptoms.

*“When we arrived there, they didn’t manage to heal her, doctors advised me to take her to traditional healers since we suspected that she had been poisoned.” (P-15, community member)*

*“Yes, I can testify that they (traditional healer) cure poison because I went to a hospital, but it wasn’t successful. Doctors also recognized it and said ‘this person must go to traditional healers in order to find out whether s/he has not been poisoned’ .“ (P-18, community member)*

Apart from using traditional medicine as an alternative treatment, a few community members took traditional medicine concurrently with convention health services and modern medicine. One woman explained that when her child had a fever, she washed the body with *capucine* (a type of plant that commonly available in the local home gardens) to decrease the body temperature, following which they took the child to a hospital to find out whether there was an infection which would cause the temperature to rise again. Another participant reported that her child had suffered from poisoning. The traditional healer recommended that she should keep using traditional medicine to cure the poison symptoms (severe pain) and meanwhile taking modern medicine given by the hospital to reduce a fever.

## Discussion

This qualitative study was conducted to explore why community members use traditional medicine in Rwanda where the UHC has been achieved. Our findings suggest that the characteristics of traditional medicine are responding to community members’ health, social and financial needs which are insufficiently met by the current UHC through providing the conventional health services. Importantly, participants used traditional medicine particularly to deal with a culture-specific illness - ‘*uburozi*’. To treat *uburozi* appropriately, the referrals from hospitals to traditional healers took place spontaneously.

One key characteristics of traditional medicine that satisfied patients’ needs was the person-centred care in the practice of diagnosis and active patient engagement in the treatment process. Previous literature reports that conventional health services which engaged patients in the healthcare decision-making resulted in better physician-patient relationships and reduction in patients’ anxiety [24]. In our research, traditional healers engaged the patients not only in the decision-making process but also the process of traditional medicine preparation. Some traditional healers explained to the patients about herbs and components of the medicine they are making. Through the treatment engagement, patients were able to build up better knowledge and understanding of the traditional medicine, which may have contributed to the improved trust with traditional healers and their treatment practices. Ozawa [25] highlighted the important role of trust in the healthcare services, such as improvement of quality of care, better patients’ cooperation with treatment and their treatment adherence. Therefore, the trust building between traditional healers and community members through better understanding of treatment components can possibly improve the quality of health service provision.

Additionally, our findings showed that traditional healers rely on listening to patients’ health complaints and their narration of illness to diagnose health problems, which requires a significant amount of time. This however is limited in the conventional health services in which medical doctors typically have limited consultation time with patients as reported in previous research [26]. The length of consultation time is related to good practitioner-patient communication which plays an important role in the quality of care [27]. Therefore, the attentive consultation with sufficient time dedicated to the patient can contribute to the improvement of healthcare services. These characteristics, including trust building through patient involvement in treatment preparation, and attentive listening of patients’ suffering, are typical for traditional medicine. With these characteristics, traditional medicine satisfies patients’ specific needs that are not sufficiently met by the current conventional health services.

The most explicit example of how traditional medicine meets the patient’s health needs, which cannot be met by the current conventional health services, was through providing treatment and care for culture-specific illness – “poisoning”. Based on our findings, it is widely believed that only traditional medicine is able to treat “poisoning” despite the existence of conventional healthcare services. Previous studies have pointed out the connections between local conceptualisation of poisoning and mistrust in the community. Burnet, in the analysis of the concepts of ‘evil’ in regard to the Rwandan genocide, summarizes that the fear of being poisoned is still very much alive in Rwanda [28]. An ethnographic study, conducted in a refugee camp where Congolese refugees settled in Uganda, indicated that “poison” is perceived as a result of living in an insecure social environment where people live in close proximity to those they are not familiar with [29]. Given these social contexts which include mistrust and insecurity, we theorize that, in some cases, “poisoning” may be attributed to the issues related to interpersonal and community relationships. Traditional healers, who are largely trusted and respected in the community and who provide social support, can possibly build up and improve the community relationships. This may alleviate the culture-specific illness symptoms which is perceived to be related to interpersonal relationship issues.

In relation to poisoning, this study importantly found that the symptoms of poisoning generally include somatization and mental illness symptoms. Previous research provides some indication of why community members may turn to traditional healers for these two illness symptoms. First, somatization may not correspond to the clinical diagnostic categories or sometimes can be medically unexplained [30]. Second, the concept of mental illness in Africa is highly determined by historical context and cultural influence rather than the conventional clinical classification [31]. In this case, traditional healers address the body-mind-spirit connections and give culturally appropriate explanations for mental illness symptoms and somatization. This provides a meaning to patients’ suffering, which is an important part of the healing process [32].

Importantly, our participants reported the collaborative referral pathway between medical doctors and traditional healers. The findings highlighted that, to establish the mutual referral systems with the ultimate aim of improving the health services, education for both traditional healers and medical doctors would be needed. In Musanze district, some traditional healers were recognised through the Traditional Healers Rwanda Association - Abavuzi Gakondo Rwanda Network (AGA-Rwanda Network). The traditional healers who were recognised through this network were provided trainings with certificates on biomedical knowledge, safety and proper use of traditional medicine at the Institute of Applied Sciences (INES) Ruhengeri [33]. One the other hand, providing training for medical doctors would be also helpful to prevent and reduce discrimination against traditional medicine. This could significantly help inform patients about treatment options and obtain appropriate treatments.

In addition to providing education to traditional healers and doctors for facilitating the referral systems, it is also important to improve the current national policy on integrating traditional medicine into the health system. The current Rwandan national policy of traditional, complementary and alternative medicine released by Rwanda Ministry of Health [34] should consider including a national health insurance for traditional medicine. In our study, traditional healers accommodated with patients’ financial needs by applying flexible payment system. Previous report has revealed that the current Rwandan national health insurance system becomes a burden for community members who have no stable cash income. Some members are categorized as wealthier than they actually are and experience difficulties in paying the premiums [35]. Our study revealed that traditional healers’ flexible payment system compensates the national health insurance system by providing traditional medicine while taking consideration of financial capacity and allowing flexible goods exchange. Such flexible payment system was part of community-based gift economy system, in which money was not necessary to gain goods and services. Thus, community members who have no stable cash income (e.g. farmers) could afford it. If it could be managed within the national health insurance system, the financial burden for patients may be be reduced. Related to this, traditional healers who charge too much, resulting in a financial burden for patients could be controlled by the national health system. This would prevent individuals from a financial catastrophe when utilising the services. Additionally, the flexible payment system within traditional healers’ practices can preserve good healer-patient relationship; by showing concern about the financial circumstances of the patients, trust can be built up. This allows patients to maintain a gift economy which embodies the spiritual power of social connection between traditional healer and patients [21]. This social connection which can build up a healer-patient trust may not be found in the fixed payment system of current conventional healthcare services; thus the policy can consider to preserve the flexible payment system when traditional medicine is integrated into the national health insurance coverage.

However, there were some safety issues reports, including the unclear expiration date of herbal medicine and vague instruction resulting in over-dosage. The insufficient instructions in the past provided by some traditional healers can be possibly attributed to the lack of regulation and standards as well as the variance of traditional healers’ knowledge and practices. Therefore, it remains important to establish an adequate regulatory framework to ensure the quality and safety controls of traditional medicine as well as for the management of traditional healers [3]. Nowadays, the importance of establishing regulatory framework is recognised through Rwanda Standards Board and Rwanda Food And Drugs Authority who handle the issues of standards and accreditation in food and pharmaceutical sectors [36, 37]. Researchers can establish collaborative networks with these governmental organisations in providing rigorous and scientific evidence to support the regulations and standardisations of traditional medicine.

Furthermore, participants reported their experience in using herbs concurrent with conventional treatments. Both adverse effects and potential greater therapeutic outcomes of herb-drug interaction have been raised in the previous research [38, 39]. Thus, there is a need for further evidenced-based research to address how to minimise the adversity of herb-drug interaction and achieve better health outcomes for patients.

Despite the useful findings and contributions as above, the findings should be considered within certain limitations. Firstly, due to the first author’s short-term stay within the community, most participants met the first author for the first time at the interview. Therefore, the first author may not have been able to build up a strong enough trust with participants to allow them to share more sensitive and culture-embedded experience which spiritual healings may involve. To minimise this impact on the study findings, the first author conducted all the interviews and observation with a fieldworker from local community (the fourth author) and had cultural and contextual inputs from other Rwandan authors. Yet, participants had different levels of relationship closeness with the data collectors (authors), which might have influenced collected data and their interpretation. The presented data need to be seen as such.

## Conclusion

Traditional healers provide treatment and care to respond to patients’ specific needs that the current conventional health services may not sufficiently address. In Rwanda, conventional health services universally cover diseases that are diagnosed by the standard of conventional medicine. However, this universal health coverage does not cover culture-specific illness as well as sufficiently respond patients’ social and financial needs arising from the health needs. Given this, it would be beneficially to integrate traditional medicine into national health systems, with adequate regulatory framework to ensure the quality and safety controls of traditional medicine.

## Data Availability

The datasets used and/or analysed during the current study are available from the corresponding author on reasonable request due to the promised confidentiality with the participants.

## Abbreviations

WHO: World Health Organisation
UHC: Universal Health Coverage
